# The Relationships between cyclin D1 Expression and Prognosis of Non-small Cell Lung Cancer

**DOI:** 10.3779/j.issn.1009-3419.2010.08.10

**Published:** 2010-08-20

**Authors:** Jiping ZHU, Like YU, Ping ZHAN, Yong SONG, Qin WANG

**Affiliations:** 1 Department of Respiratory Medicine, Nanjing Chest Hospital, Nanjing 210029, China; 2 Department of Respiratory Medicine, Jinling Hospital, Nanjing University School of Medicine, Nanjing 210001, China; 3 Department of Respiratory Medicine, 81 Hospital of PLA, Nanjing 210002, China

**Keywords:** Lung neoplasms, cyclin D1, Prognosis, Immunohistochemistry

## Abstract

**Background and objective:**

cyclin D1 is a member of the cyclin family, and it has been proven that it plaied an important role in tumorigenesis, invasion and metastasis. We performed a retrospective study on the cyclin D1 expression in non-small cell lung cancer (NSCLC) according to the clinical characteristics.

**Methods:**

One hundred fifteen postsurgical NSCLC patients were investigated. Immunohistochemistry was used to evaluate the cyclin D1 expression.

**Results:**

Overall survival was significantly lower in patients with cyclin D1-high expression of tumors than those with cyclin D1 low expression of tumors (χ^2^=5.132, *P*=0.023). In early stage patients (stage Ⅰ, Ⅱ), the overall survival was significantly lower in patients with cyclin D1-high expression of tumors than those with cyclin D1-low expression of tumors (χ^2^=6.863, *P*=0.009). cyclin D1 status (hazard ratio=0.630;*P*=0.035), differentiation (hazard ratio=0.399; *P* < 0.001), and pTNM (hazard ratio=1.576; *P* < 0.001) to be independent prognostic factors for NSCLC patients. Specifically, the cyclin D1 status (hazard ratio=0.188;*P*=0.008) was a significant prognostic factor for patients with stage Ⅰ NSCLCs.

**Conclusion:**

cyclin D1 expression is an independent prognosis factor for postoperative patient in stage Ⅰ, Ⅱ NSCLCs.

## Background

Lung cancer is a leading cause of death due to cancer (1.4 million deaths/each year) ^[1]^. Thereinto, non-small cell lung cancer (NSCLC) accounts for 85%. But at present, chemotherapy, radiotherapy, and surgery are the first priorities in the medical treatment. The choice of the treatment is determined by TNM (tumor-node-metastasis) stage^[2]^. Unfortunately, the effect of treatment for NSCLC is far from perfect. The 5-year survival rates for stage Ⅰ, Ⅱ, Ⅲ are 47%, 26% and 8.4% respectively^[3]^. Therefore, much importance should be attacked to the further clarification of the mechanism of tumor biology and its pathogenesis, and the study on the factors which will affect the prognosis, in expectation of giving more pertinent and timely treatment, improving the prognosis and prolonging the survival period of NSCLC patients. The cyclin is an important protein to regulate the cell cycle. At different stages of cell mitosis, the components of cyclin family intergrate with cyclin-dependent kinases (CDK), and form a complex acting as a regulatory subunit of CDK. cyclin D1 is an indispensable regulatory protein to cell cycle in G_1_/S transition. It forms a complex with CDK4 or CDK6, which phosphorylates retinoblastoma protein which is involved in STAT5A-regulated transcription. Overexpression of cyclin D1 could alter the process of the cycle and induce excessive proliferation of cell or even tumor^[4]^. The current research shows that down-regulating the expression of cyclin D1 could restrain proliferation of tumor cells^[5]^. Another study found cancers with higher indexes of cyclin D1 expression have the stronger capacity to metastasize^[6]^. Therefore, there comes the hypothesis that the expression of cyclin D1 in lung cancer cell could impact the survival time of lung cancer patients. The aim of this study is to evaluate the expression of cyclin D1 in lung cancer tissues by means of immunohistochemistry, and to analyze the relationship between the amount of tumor staining for cyclin D1 and overall survival.

## Materials and Methods

### Patients

A total of 115 tumor specimens were obtained from the patients who had undergone surgeries at Nanjing Chest Hospital and No. 81 Hospital of PLA from January 2001 to December 2005. None of the in-patients with NSCLC received any chemotherapy or radiotherapy before their surgeries, and all of them had surgeries as their first line of management. The patients' characteristics are presented in Table 1. The patients included 87 males and 28 females, aged 17-80 years (mean 59.7 years). According to the classification of the World Health Organization (WHO), the specimens were classified into 63 (54.8%) adenocarcinomas (of that, 13 tumors were BAC), 40 (34.8%) squamous cell carcinomas, 12 (10.4%) others (large cell carcinomas, adenosquamous carcinomas and carcinoid). The p-Stage and pN-Stage were determined according to the guidelines of the American Joint Committee on Cancer^[7]^. 105 (91.3%) patients received postoperative adjuvant chemotherapy by the third generation of platinum-based regimens. Inclusion criteria for this study were surgical complete resection of the tumor (resection margin microscopically free of tumor cells); survived for more than 3 months after surgery; not dying of causes other than lung cancer within 5 years after surgery. The patients' clinical records and histopathological diagnoses were fully documented. The date of last followup was March 21, 2008.

**1:** Distribution of 115 non-small cell lung cancer patients according to cyclin D1 status

Variables	No. of patients (%)	cyclin D1		*P*
		Low	high	
All patients	115(100)	60	55	
Gender				0.791
Male	87(75.7)	46	41	
Fmale	28(24.3)	14	14	
Age				0.987
< 60	44(38.3)	23	21	
≥60	71 (61.7)	37	34	
Smoking				0.181
Non-smoker	47(40.9)	21	26	
Smoker	68(59.1)	39	29	
Size of tumor				0.169
≤3 cm	32 (27.8)	20	12	
> 3 cm	83 (72.2)	40	43	
T-stage				0.277
T1, T2	89 (77.4)	44	45	
T3, T4	26(22.6)	16	10	
Histology				0.058
Squamous cell carcinoma	40(34.8)	24	16	
Adenocarcinoma	63 (54.8)	27	36	
Other	12(10.4)	9	3	
N-stage				0.418
N0	42 (36.5)	24	18	
N1+N2	73(63.5)	36	37	
p-TNM stage				
Ⅰ	36 (31.3)	20	16	0.130
Ⅰa	13	10	3	0.083^*^
Ⅰb	23	10	13	
Ⅱ	27 (23.5)	n	16	
Ⅲ	41 (35.7)	20	21	
Ⅳ	11 (9.6)	9	2	
Bronchoalveolar carcinoma				0.293
Yes	13(11.3)	5	8	
No	102(88.7)	55	47	
Differentiation				0.717
Well/Moderate	67 (58.3)	26	22	
Poor	48(41.7)	34	33	
^*^Fisher's Exact Test.				

### Immunohistochemical staining

Antibodies for immunohistochemical analyses were obtained. As follows: rabbit anti-human cyclin D1 monoclonal antibody (working solution, ZA-0101, Zhongshan Goldenbridge Biotechnology, Beijing, China), rabbit antihuman caveolin-1 polyclonal antibody (1:300, N-20: sc-894, Santa Cruz Biotechnology, Santa Cruz, USA). Resected specimens were fixed in 10% formalin, and paraffin-embedded blocks were prepared. Next, 4 μm thick sections were cut from the specimens and placed on slides coated with poly-L-lysine. Immunohistochemical staining was performed by using the EnVision two-step procedure immunohistochemical method (EnVision Detection Kit, Peroxidase/DAB, Rabbit/Mouse, DAKO, Denmark), and the operations were carried out strictly following the manufacturer's instructions. In brief, sections were routinely deparaffinized with xylene and rehydrated in decreasing concentrations of alcohol. Antigen retrieval was done by placing the specimen in EDTA retrieval agent (ZLI-9066_ZLI-9067, Zhongshan Goldenbridge Biotechnology, Beijing, China) at pH8.0, and autoclaved at 12 ℃ for 2 min to allow for fixing. The sections were washed in phosphate-buffered saline (PBS) buffer (pH7.6), then the sections were incubated overnight at 4 ℃ in a moist chamber with the antibody. After washing the sections in PBS three times for 5 min, they were treated for 30 min at room temperature in ChemMate EnVision+/HRP (DAKO, Denmark). Subsequently, the sections were washed with PBS, and DAB colorization was applied, followed by application of diaminobezidine (DAB) solution (ChemMate EnVision+/DAB, DAKO, Denmark) until color developed. Staining was monitored under a bright-field microscope, and the reaction was stopped by washing with distilled water. The sections were then counterstained with hematoxylin; dehydrated in increasing concentrations of alcohol, and coversliped with neutral gummy.

### Immunohistochemial staining evaluation

The slides were independently reviewed by two of the authors (P.Z. and Q.W.) who had no knowledge of the patients' clinicopathological status. If the discrepancies existed between the reviewers, a consensus judgment was reached through discussion. The proportion of staining tumor cells in each selected field was determined by counting individual tumor cells at randomly chosen 4 high magnification (×400) fields by using a light microscope (Model CX31RTSF, Olympus, Tokyo, Japan). The immunoreactivities were graded as (-), (+), (++), (+++) according to the percentage of the positive tumor cells: (-) represents zero or less than 5% positive tumor cells; (+) represents 5%-25% positive tumor cells; (++) represents 25%-50% positive tumor cells; and (+++) represents the strongest staining with more than 50% positive tumor cells.

For cyclin D1^[6]^, the samples of breast cancer (whose cells had positive nuclear staining for cyclin D1) were referred to as a positive control, while the negative controls were included by omission of the primary antibody. More than 5% of tumor cells (+) showed definitive nuclear positivity, which was considered cyclin D1 high expression.

### Statistical analysis

Statistical analysis was performed using the SPSS for Windows v.13.0 package program. *Chi-square* test was used for comparison of data between groups. Overall survival (OS) was calculated from the day of surgery to the date of last followup or the date of death. While death of no recurrence patients or survival at last follow-up date is considered to be censored. OS curves were computed according to the method of *Kaplan-Meier*. To assess the independent value of different variables on survival in the presence of other variables, multivariate analysis was carried out using the *Cox* proportional hazards model. Analysis was performed using backward *Wald's* criteria. A value of *P* < 0.05 was accepted as statistically significant, and all tests were two-sided.

## Results

### Follow-up

Median time of follow-up was 22 months (rangeing from 3 to 83 months). 30 cases were censored, accounting for 26.1% of all the patients.

### cyclin D1 expression in NSCLCs

cyclin D1 staining featured a heterogeneous nuclear staining pattern (Fig 1) in tumor cells. 55 cases were high expression, accounting for 47.8% of all the 115 cases with NSCLCs. 60 cases were low expression, with a percentage of 52.2%. There is no significant difference in the cyclin D1 expression in relation to gender (*P*=0.830), smoke habit (*P*=0.190), tumor status (*P*=0.373), nodal status (*P*=0.444), histology (*P*=0.058), pathologic stage (*P*=0.130) or differentiation (*P*=0.850).

**1:**
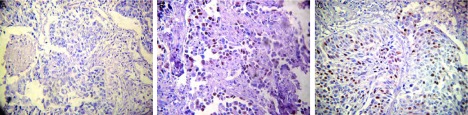
Immunostaining of NSCLC in serial sections. A: negative contrast; B: adenocarcinoma with positive expression of cyclin D1; C: squamous cell carcinoma with positive expression of cyclin D1.

### Overall survival of NSCLC patients in relation to cyclin D1 status

The overall survival was significantly lower in patients with cyclin D1-high expression of tumors than in those with cyclin D1-low expression of tumors (*P*=0.023) (Fig 2a, Tab 2). In early stages (stage Ⅰ, Ⅱ), the overall survival of the patients with cyclin D1-high expression of tumors was significantly lower than those with cyclin D1-low expression of tumors (*P*=0.009) (Fig 2b, Tab 2). Multivariate regression analysis based on the *Cox* proportional hazards regression model demonstrated that the cyclin D1 status (hazard ratio=0.630;*P*=0.035), differentiation (hazard ratio=0.399; *P* < 0.001), and pTNM (hazard ratio=1.576; *P* < 0.001) were independent prognostic factors for NSCLC patients (Tab 3). Especially the cyclin D1 status (hazard ratio=0.188;*P*=0.008) was a significant prognostic factor for patients with stage Ⅰ NCSLC (Tab 4).

**2:**
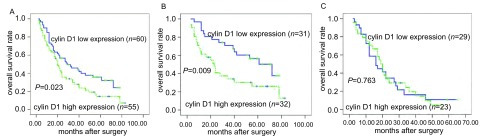
Overall survival of 115 NSCLC patients in relation to cyclin D1 status. A: in total of 115 NSCLC patients; B: in 63 patients with stage Ⅰ, Ⅱ NSCLCs; C: in 52 patients with stage Ⅲ, Ⅳ NSCLCs.

**2:** Survival analysis of 115 patients with non-small cell lung cancer

Parameter	*n*	Deaths	Survival time (month)			*Log-rank* test	
			Mean (95% CI)	Median (95% CI)		*X*^2^	*P*
All pateints	115	85	35.27(29.91-40.63)	23.00(17.01-28.91)		5.132	0.023
cyclin D1 low expression	60	39	39.86 (32.50-47.23)	29.00(12.07-45.93)			
cyclin D1 high expression	55	46	29.02 (22.19-35.869)	20.00 (15.97-24.05)			
Stage Ⅰ, Ⅱ	63	39	44.75 (36.96-52.54)	40.00 (23.27-56.73)		6.863	0.009
cyclin D1 low expression	31	15	53.45 (43.90-63.00)	72.00 (45.84-98.16)			
cyclin D1 high expression	32	24	34.39 (23.94-44.84)	23.00 (1770-28.30)			
Stage Ⅲ, Ⅳ	52	46	22.27 (17.67-26.88)	17.00 (13.47-20.53)		0.091	0.763
cyclin D1 low expression	29	24	22.95 (16.08-29.82)	16.00(9.41-22.59)			
cyclin D1 high expression	23	22	21.52(15.31-27.73)	19.00 (15.48-22.52)			
CI: confidence interval.							

**3:** Multivariate regression analysis in predicting survival of 115 patients with NSCLC

	B	*P*	Hazard ratio	95% CI
cydin D1 status	-0.461	0.035^※^	0.630	0.411-0.967
Differentiation	-0.920	< 0.001	0.399	0.248-0.641
pTNM	0.455	< 0.001	1.576	1.255-1.980
B: partial regression coefficient; ^※^: low expression versus high expression.				

**4:** Multivariate regression analysis in predicting survival of 115 patients with NSCLC

	Stage Ⅰ			Stage Ⅱ			Stage Ⅲ			Stage Ⅳ	
	Hazard ratio	*P*		Hazard ratio	*P*		Hazard ratio	*P*		Hazard ratio	*P*
	(95% CI)			(95% CI)			(95% CI)			(95% CI)	
cyclin D1 status											
Low	0.188 (0.055-0.650)	0.008		0.587 (0.223-1.550)	0.282		0.877 (0.377-2.403)	0.762		3.362 (0.035-322.508)	0.603
High											
Differentiation											
Well/Moderate	0.265 (0.091-0.772)	0.015		0.325 (0.118-0.898)	0.030		0.334(0.152-0.730)	0.006		0.655 (0.014-30.822)	0.830
Poor											

## Discussion

Many researches showed that intratumoral cyclin D1 levels were correlated with the outcome of prognosis. Brücher BL *et al* found, low cyclin D1 levels experienced significantly less frequent recurrence of the tumor, and there was a significant difference in the recurrence-free interval^[9]^. Jaworska *et al* evaluated cyclin D1 levels of 47 specimens of resected oral and lip squamous cell carcinoma by immunohistochemistry and their pertinence with survival time of patients. The findings indicated that lower expression of cyclin D1 was correlated with longer disease free survival^[10]^. Rudas *et al* used immunohistochemistry to assess the expressions of cyclin D1 in surgical specimens from patients with breast carcinomas and colorectal cancers that received adjuvant chemotherapy by Tamoxifen, and patient survival. They found that the overall survival and Relapse-free survival of patients with positive cyclin D1 were shorter, compared with the patients with negative cyclin D1^[11]^. García *et al* used real-time PCR to examine the cyclinD1 mRNA in plasma of patients with breast cancers. They observed poor outcomes in patients with the presence of cyclinD1 mRNA in plasma among good-prognosis group (such as negative vascular invasion). Furthermore, the presence of cyclin D1 mRNA was correlated with relapse after surgery and insensitivity to Tamoxifen^[12]^. In the researches mentioned above, without exception, observed specimens are completely resected tumor. The main factor influencing survival time was recurrence. Also, regarding the lung cancer cases of completely resected stage Ⅰ, the rate of recurrence after surgery is 25%-50%. It's probably because of occult extensiver disease undetected by traditional method at the time of surgery, including local and distant metastasis^[13]^. A *meta*-analysis indicated that 20%-70% stage Ⅰ NSCLC patients were found micrometastasis in lymph nodal. The 3-, 5-years overall survival rate for positive patients was worse than negative patients^[14]^. cyclin D1 may play a leading role in mediating invasion and metastasis of cancer cells^[15]^. Parra *et al* found cyclin D1 expression of non-metastatic adenocarcinomas was lower than metastatic adenocarcinomas. *Kaplan-Maier* analysis revealed patients with higher cyclin D1 expression were significantly shorter (*P*=0.04) ^[6]^. Another study indicated that cyclin D1 promotes cellular motility through inhibiting ROCK signaling and repressing the metastasis suppressor TSP-1^[16]^. Luo *et al* recently reported that Twist protein promoted the migration, invasion, and metastasis of the gastric cancer cells. Furthermore, overexpression of Twist promoted the expression of cyclin D1, while suppression of Twist inhibited the expression of cyclin D1^[17]^. It is obvious that high expression of intratumoral cyclin D1 not only directly promotes metastasis, but it is also an exhibition of activity of other factors related to promoting metastasis. It was presumed that cancer cells with higher expression of cyclin D1 have stronger capacity for metastasis and earlier occurrence of micro metastasis than those with lower expression of cyclin D1. Despite the complete resection of the primary tumor, the focus of micrometastasis is still present as a potential cause of recurrence, and finally influences the prognosis. Our study revealed that high expression of cycinD1 was associated with a poor prognosis in early stage NSCLC patients. The level of cyclin D1 could be a useful index to distinguish patients, whose clinical outcome is poor, at stage Ⅰ of NSCLCs' complete resection. Whether high expression of cyclin D1 is an index which can guide the postoperative adjuvant chemotherapy needs much more researches.
